# Maize Yield and Economic Response to Integrated Organic and Nitrogen Fertilization in Gaza Province, Mozambique

**DOI:** 10.1002/pei3.70184

**Published:** 2026-07-13

**Authors:** Manuel Mulhuli Sitoe, Hercidio Jaime Tandane, Ivo Marino Domingos, Olga Mário Chaguala

**Affiliations:** ^1^ Agriculture Research Institute of Mozambique South Regional Office Chókwè City Gaza Province Mozambique

**Keywords:** economic analysis, integrated fertilization, maize yield, Mozambique, nitrogen fertilizer, organic manure

## Abstract

Low maize yields in Mozambique remain a major constraint to food security, primarily due to soil nutrient depletion and limited access to affordable fertilizers among smallholder farmers. Integrated nutrient management, combining organic and mineral inputs, offers a promising approach to improve both productivity and soil health. This study evaluated the agronomic performance and economic viability of combining poultry and cattle manure with urea (mineral nitrogen) in maize production in Gaza province, Mozambique. A 3 × 3 × 3 factorial experiment arranged in a randomized complete block design evaluated three rates of cattle manure (0, 5, and 10 t·ha^−1^), poultry manure (0, 5, and 10 t·ha^−1^), and nitrogen fertilizer (0, 40, and 80 kg N·ha^−1^). Results showed that integrated treatments significantly increased plant height from 177 to 217 cm and grain yield from 3.04 to 5.23 t·ha^−1^ compared with the unfertilized controls. Soil organic matter increased from 1.60% in the control to 3.78% under 5 t·ha^−1^ cattle manure. The best agronomic performance was achieved by integrating poultry manure with 40–80 kg N·ha^−1^. Economically, the sole application of 5 t·ha^−1^ poultry manure provided the highest marginal rate of return (MRR > 1000%), indicating it as the most cost‐effective option. These findings demonstrated that integrated organic and mineral fertilization enhances maize yield and farm profitability, offering a practical solution for improving smallholder productivity in semi‐arid regions. Further multi‐season studies are recommended to validate longer‐term effects.

## 1 Introduction

Maize ( *Zea mays* L.) is one of the most important staple crops in sub‐Saharan Africa, providing food security and income for millions of smallholder farmers (Badu‐Apraku et al. 2017). In Mozambique, maize is the most widely cultivated food crop, occupying nearly 40% of the total arable land and contributing significantly to household food security and national grain production (Leonardo et al. 2015). In regions such as Gaza province, maize is predominantly produced under rainfed conditions and often on nutrient‐depleted soils (Nhantumbo et al. 2021; Tandane et al. 2026). The crop's adaptability to diverse agroecological zones and its relatively short growing cycle make it especially important in areas affected by climate variability (Zeleke et al. 2023). Despite its critical role, average maize yields in Mozambique remain low, typically ranging from 1.0 to 1.5 t·ha^−1^, well below the potential yield of 5 to 7 t·ha^−1^ achievable under improved management (Amaral et al. 2020; Nhantumbo et al. 2021). This persistent yield gap stems from a combination of biophysical and socioeconomic constraints, including limited access to inputs, poor agronomic practices, soil degradation through nutrient mining and loss of soil organic matter, and climate stress resulting from recurrent droughts and increasingly variable rainfall. Of these factors, declining soil fertility, particularly nitrogen deficiency, is widely recognized as a primary limitation to maize productivity in smallholder systems (Biramo 2018; Sommer et al. 2013).

Nitrogen is essential for vegetative growth and grain formation, yet the use of mineral fertilizers such as urea, ammonium nitrate, and calcium ammonium nitrate remains limited in many regions due to their high cost, poor distribution, and lack of technical support for proper application (Guo et al. 2026; Naz and Sulaiman 2016). Consequently, most farmers apply fertilizers at suboptimal rates, often less than 10 kg·ha^−1^, which is insufficient to meet crop nutrient demands and leads to further soil nutrient depletion (Vanlauwe et al. 2023). Locally available organic resources such as cattle and poultry manure offer sustainable alternatives, supplying not only nitrogen but also phosphorus, potassium, and organic carbon that help maintain soil structure and microbial activity (Kacprzak et al. 2023; Rayne and Aula 2020; Thobane et al. 2025). However, nutrient release from organic inputs is typically slow and may not align with crop demand due to high carbon‐to‐nitrogen ratios (C:N) and variable decomposition rates (Shaji et al. 2021). As such, sole application of manure often fails to meet short‐term nutrient needs, particularly in low‐input farming systems (Tiemann and Douxchamps 2023).

Integrated Soil Fertility Management (ISFM), which promotes the combined use of organic and inorganic amendments (e.g., manure, compost, or crop residues) and mineral fertilizers at site‐specific and often optimized rates, has gained increasing attention as a practical and scalable solution to improve nutrient use efficiency, crop productivity, and profitability in sub‐Saharan Africa (Doldt et al. 2023; Mugwe et al. 2019; Vanlauwe et al. 2015). ISFM not only enhances nutrient synchrony but also improves soil resilience and long‐term fertility, key priorities in regions like the Gaza province where rainfall variability and low fertilizer use persist. While integrated nutrient strategies have shown success across various African agroecosystems, limited evidence exists for semi‐arid environments in southern Mozambique, especially regarding the combined effects of poultry and cattle manure with nitrogen fertilizer on maize yield, soil properties, and economic returns. Moreover, field based evaluations under realistic smallholder conditions are needed to inform context‐appropriate fertilizer recommendations.

Therefore, the aim of this study was to evaluate the agronomic and economic response of maize to integrated applications of cattle and poultry manure with mineral nitrogen fertilizer in Gaza province, south of Mozambique. Specially, the study assessed treatments effects on grain yield, plant height, biomass accumulation, soil fertility indicators, and marginal rate of return. The remainder of this paper is organized as follows: Section 2 presents the study area and methodology; Section 3 outlines the results and discusses the findings considering previous literature and local agronomic contexts; and Section 4 concludes with implications for practice and future research.

## 2 Material and Methods

### 2.1 Study Area

The study was conducted at the Chókwè Agriculture Research Station, located in Chókwè district, Gaza province, in south Mozambique. The Research Station is situated near the Limpopo River, at approximately 24°52′S, 33°00′E, and lies 33 m above sea level. The district is characterized by a semi‐arid climate, with mean annual temperatures ranging from 22°C to 26°C, and annual rainfall typically between 500 and 700 mm (Melo et al. 2019). The dominant soil types in the study area are alluvial and red clay soils (Saveca 2016).

### 2.2 Treatment and Experimental Design

The experiment was conducted from April to August 2022 using a randomized completely block design in a 3 × 3 × 3 factorial arrangement. The study included three factors (see Supporting Information): beef cattle manure (Factor A), poultry litter (Factor B), and mineral nitrogen (Factor C), each applied at three levels. Beef cattle manure was applied at 0 t·ha^−1^ (A1), 5 t·ha^−1^ (A2), and 10 t·ha^−1^ (A3). Poultry litter was applied at 0 t·ha^−1^ (B1), 5 t·ha^−1^ (B2), and 10 t·ha^−1^ (B3). Mineral nitrogen, in the form of urea containing 46% N, was applied at 0 kg N·ha^−1^ (C1), 40 kg N·ha^−1^ (C2), and 80 kg de N·ha^−1^ (C3). This factorial scheme resulted in 27 treatment combinations, each replicated three times, giving a total of 81 experimental plots. Each plot comprised four rows, each 4 m in length, with spacing of 0.80 m between rows and 0.20 m between plants within the row. The central two rows were used as the net plot, and the total net experimental area was 2038,4 m^2^. The maize variety used was Matuba, sown manually. The organic manure was applied as basal fertilization by incorporating the respective quantities directly into the planting furrows prior to sowing. The application of mineral nitrogen was carried out in two equal splits, corresponding to 20 and 20 kg N·ha^−1^ for the 40 kg N·ha^−1^ treatment, and 40 to 40 kg N·ha^−1^ for the 80 kg N·ha^−1^ treatment, applied at 21 and 40 days after sowing, respectively. The chemical composition of the cattle and chicken manure used in the experiment is presented in Table 1.

**TABLE 1:** Chemical composition of cattle and poultry manure used in the experiment.

Description	Cattle manure	Poultry manure	Method of analysis
pH‐H_2_O	7.59	9.19	Potentiometer
EC 1:2.5 (mS/cm)	0.00864	0.0032	Conductivity meter
P (mg/100 g soil)	1.55	33.29	Olsen
K (mg/100 g soil)	1.02	1.39	Flame photometer
Total N (%)	3.885	1.778	Kjeldahl
O.M. (%)	69.98	72.03	Walkey&Black
ECC (mg/100 g soil)	18.50	30.40	Percolation of AcNH4

### 2.3 Soil Sampling and Analysis

Soil sampling was carried out in two phases: the first, before land preparation, and the second, after harvest. In the first phase, a single composite soil sample was collected using the zigzag method at a depth of 0–30 cm across the experimental area. After harvest, soil samples were taken per treatment plot at the same depth. All samples were analyzed individually and then averaged per treatment. The laboratory analysis included determination of total nitrogen, organic matter, soil pH‐H_2_O, and electrical conductivity. All determinations were based on the individual treatment samples and performed according to established laboratory protocols (Sharma et al. 2018; Wogi et al. 2021). Soil moisture was monitored every 21 days using a soil probe. Samples were collected from the 0–30 cm soil layer, one per treatment plot. Moisture content was determined using the gravimetric method, where the fresh soil mass was recorded, and then the sample was oven‐dried at 105°C for 48 h before recording the dry mass. Soil moisture was calculated using the formula:

(1) $$H=\frac{\left(\mathrm{Mh}-\mathrm{Ms}\right)}{\mathrm{Ms}}\times 100$$

where *H* represents the soil moisture content (%), *Mh* is the mass of moist soil (g), and *Ms* is the mass of dry soil (g).

### 2.4 Plant Growth and Yield Evaluation

Grain yield was assessed by harvesting all plants from the net plot area and weighing the grain immediately. Grain moisture at the time of weighing was determined using a digital moisture meter. The yield was corrected to a standard moisture content of 12.5% and expressed in tons per hectare using the equation proposed by (Bidyarani et al. 2016).

(2) $$R=\mathrm{Pg}*\frac{100-H.\;\text{real}}{100-12.5}*\frac{10}{\mathrm{Au}}$$

where *R* is the grain yield (t·ha^−1^), *Pg* is the weight of grain (kg), *H* is the actual moisture content of the grain at weighing (%), and *Au* is the net plot area (m^2^). Plant height was measured from the first basal node to the base of the male inflorescence using a wooden ruler. Plant height was measured from the first basal node to the base of the male inflorescence using a wooden ruler. Measurements were taken from all plants within the net plot, and the mean plant height per treatment was calculated.

Dry biomass was measured by collecting aerial parts of maize plants from each treatment plot, immediately weighing them, and then air‐drying them under sunlight for approximately 2 weeks. After drying, samples were reweighed. Biomass was calculated using the formula:

(3) $$B=\frac{\mathrm{Pb}*10^{-3}}{\mathrm{Au}*10^{-4}}$$

where *B* is the dry biomass (t·ha^−1^), *Pb* is the dry weight (g), and *Au* is the net plot area (m^2^), as described by Fixen et al. (2015).

### 2.5 Soil Fertility Indicators

The effect of the treatment on soil fertility was assessed through laboratory determination of total nitrogen and organic matter before and after the experiment. The percentage values obtained for each treatment were subjected to statistical analysis to evaluate treatment effects on soil chemical properties.

### 2.6 Statistical Analysis and Economic Analysis

Data from all measured variables were subjected to analysis of variance (ANOVA) using the statistical package software Statistix version 8.0 (Ibrahim and Abdullahi 2023; Oluwaseun 2017). Where significant treatment effects were observed (*p* ≤ 0.05), the means were compared using Tukey's test at a 5% level of probability. Both isolated and combined effects of organic and mineral fertilization were evaluated across treatments.

#### 2.6.1 Marginal Rate of Returns Analysis

In addition to the agronomic evaluation, an economic evaluation was carried out using partial budgeting to assess the profitability of each treatment. The MRR was used to determine the return on the additional investment made in each treatment compared to the control. This involved comparing the increase in net profits with the additional variable costs incurred. A treatment was considered economically viable when its MRR exceeded 100%, which is generally regarded as the minimum acceptable rate of return for smallholder farmers in developing countries (Evans 2005).

## 3 Results and Discussion

### 3.1 Soil and Manure Chemical Properties

The chemical composition of the cattle and poultry manure used in the experiment, along with baseline and posttreatment soil characteristics, is presented in Table 2. Both organic manures showed a notably high organic matter (O.M) content, with 69.98% in cattle manure and 72.03% in poultry manure, highlighting their strong potential as soil fertility enhancers. These high O.M values are consistent with nutrient‐rich organic residues often used to improve soil structure and microbial activity (Coonan et al. 2020; Siedt et al. 2021). The C:N ratio differed substantially between the two manure types. Cattle manure exhibited a lower C:N ratio (10.46) compared to poultry manure (23.52), suggesting a faster mineralization rate and more immediate nutrient release from the cattle manure. A low C:N ratio is generally associated with net nitrogen mineralization, whereby soil microorganisms release plant‐available nitrogen rather than immobilizing it during decomposition. This process can enhance nutrient cycling, improve nitrogen availability, and contribute to better soil fertility by increasing the availability of essential nutrients to crops. In contrast, the higher C:N ratio of poultry manure may initially immobilize nitrogen but could offer more sustained nutrient release over time (Qiu et al. 2021).

**TABLE 2:** Chemical characteristics of the manures and soil samples used in the experiment.

Description	Treatment	O.M (%)	C/N	pH‐H_2_O	EC (mS/cm)	Total N (%)	Moisture (%)
Manure composition	Cattle manure	69.98	10.46	7.59	0.00864	1.778	—
	Poultry manure	72.03	23.52	9.19	0.0032	3.885	—
Initial soil condition	Composite sample	3.04	—	7.30	0.35	0.10	—
Soil condition postharvest per treatment applied	C1B1A3	3.35	—	7.52	0.30	0.08	24.71
	C1B1A2	3.78	—	7.54	0.29	0.09	24.11
	C1B3A1	2.00	—	7.30	0.44	0.15	24.06
	C1B2A1	2.64	—	7.56	0.28	0.11	27.1
	C1B1A1 (control)	1.60	—	7.52	0.22	0.06	25.21

The initial organic matter in the soil was 3.04%, classified as moderate according to tropical soil fertility benchmarks (Sanchez et al. 2003). Posttreatment O.M levels varied significantly among treatments, ranging from 1.60% in the unfertilized control (C1B1A1) to 3.78% in the treatment receiving 5 t·ha^−1^ cattle manure (C1B1A2). These results indicate that even within a single growing season, organic input began to enhance soil O.M content. Treatments with either poultry or cattle manure consistently outperformed mineral‐only or unfertilized treatments in terms of O.M enrichment, reflecting the short‐term impact of organic amendments on soil quality. Regarding total nitrogen (N), postharvest values remained low across all treatments, generally below 0.15%, indicating low soil nitrogen status according to fertility thresholds commonly reported for tropical and sub‐Saharan African soils (Folmer et al. 1998; Vanlauwe et al. 2017, 2023). Nonetheless, modest increases in total N were observed in treatments receiving organic inputs. For instance, the C1B1A2 treatment (5 t·ha^−1^ cattle manure) recorded a total N of 0.09%, compared to 0.06% in the control. Similarly, poultry manure treatments also resulted in improved total N content (e.g., C1B3A1: 0.15%). These increments, though moderate, reflect early signs of nutrient release and microbial activity enhancement due to manure addition. The limited improvement in total nitrogen may be attributed to the short duration of the experiment and the slow decomposition rate of organic residues. As noted by Thomas et al. (2019), the full benefits of organic amendments on soil nutrient status often become evident only after multiple cropping cycles, when mineralization has advanced and stable humic substances begin to accumulate.

### 3.2 Maize Yield Response

The analysis revealed that organic fertilizer applied alone, whether poultry or cattle manure, did not significantly increase maize grain yield during the first cropping season (Table 3). This finding is consistent with earlier studies reporting limited short‐term yield benefits from organic amendments when used in isolation (Nie et al. 2024; Wei et al. 2016; Wortman et al. 2017). The primary reason is the slow mineralization rate of organic materials, which often delays the release of plant‐available nutrients such as nitrogen, especially in low moisture or low biological activity soils. In contrast, integrated treatments combining organic manure with mineral nitrogen fertilizer resulted in significantly higher grain yields compared to the unfertilized control. The highest yield (5.23 t·ha^−1^) was recorded in the treatment combining 80 kg N·ha^−1^ with 5 t·ha^−1^ poultry manure, a combination that performed significantly better than the control plot (3.04 t·ha^−1^) and treatment with only organic or mineral fertilizer alone. This outcome reinforces the concept of nutrient synergy, where the fast release nitrogen from urea complements the slower, sustained release from organic inputs, improving overall nutrient uptake efficiency.

**TABLE 3:** Best fertilizer combinations affecting maize yield.

Treatment	Maize grain yield (t·ha^−1^)
C3B2A1	5.23a
C3B3A1	5.22a
C3B1A2	4.59ab
C3B1A3	4.39ab
C3B1A1	3.88ab
C1B1A1	3.04b

*Note:* The small letters (a, ab, b) indicate the statistical differences among the treatments.

Interestingly, treatments combining moderate nitrogen input (40 kg N·ha^−1^) with organic manure can enhance nutrient efficiency and reduce dependency on costly mineral fertilizers, an important consideration for resource constrained smallholder farmers in semi‐arid regions (Iqbal et al. 2019; Sileshi et al. 2019). These findings underscore the potential of ISFM strategies to optimize productivity while minimizing input costs. By improving nutrient synchrony and enhancing soil organic matter, ISFM approaches contribute not only to higher yields but also to long‐term sustainability of smallholder farming systems in southern Mozambique and similar agroecological zones.

### 3.3 Effect of Mineral, Organic, and Integrated Fertilization on Maize Plant Height

Maize plant height was significantly influenced by the different fertilization strategies (Table 4). A clear positive trend was observed with increasing rates of mineral nitrogen, with the tallest plants (210.67 cm) recorded in the 80 kg N·ha^−1^ treatment (C3B1A1). In contrast, the lowest plant height (177.00 cm) was observed in the unfertilized control (C1B1A1). This result underscores the essential role of nitrogen in promoting vegetative growth, especially leaf and stem development, due to its function in the synthesis of chlorophyll, proteins, and enzymes (Fathi 2022; Zaidi et al. 2017). Treatments with organic manure alone, whether poultry or cattle, resulted in insignificant plant heights. Although the differences between manure types were not statistically significant, both produced plant heights significantly greater than the control. The highest values among organic‐only treatments were achieved with 10 t·ha^−1^ poultry manure (203.00 cm) and 5 t·ha^−1^ cattle manure (201.33 cm). These results highlight the potential of organic input to enhance plant growth, even in the short term, likely due to improved soil physical conditions and gradual nutrient release following microbial decomposition (Ahmad et al. 2016).

**TABLE 4:** Maize plant height under mineral, organic and integrated fertilization treatments.

Treatment	Fertilization type	Rate applied	Plant height (cm)
C3B1A1	Minera N	80 kg N·ha^−1^	210.67a
C2B1A1	Minera N	40 kg N·ha^−1^	191.00ab
C1B1A1	Control	0.00	177.00b
**Organic**			
C1B3A1	Poultry manure	10 t·ha^−1^	203.00a
C1B2A1	Poultry manure	5 t·ha^−1^	199.00ab
**Organic**			
C1B1A2	Cattle manure	5 t·ha^−1^	203.33a
C1B1A3	Cattle manure	10 t ·ha^−1^	197.67ab
**Integrated fertilization**			
C2B3A1	Poultry manure	40 kg N + 10 t·ha^−1^	217.00a
C2B1A2	Cattle manure	40 kg N + 5 t·ha^−1^	210.00ab
C2B1A3	Cattle manure	40 kg N + 10 t·ha^−1^	208.67ab
C2B2A1	Poultry manure	40 kg N + 5 t·ha^−1^	206.67ab

*Note:* *p* value = 0.1704; CV = 0.44. The small letters (a, ab, b) indicate the statistical differences among the treatments.

Notably, integrated treatments combining 40 kg N·ha^−1^ with either poultry or cattle manure yielded the tallest plants overall. The highest plant height (217.00 cm) was observed in the treatment combining 40 kg N·ha^−1^ with 10 t·ha^−1^ poultry manure (C2B3A1). All integrated treatments outperformed both the control and the mineral‐only treatment at 40 kg N·ha^−1^ (C2B1A1, 191.00 cm), suggesting a synergistic effect between mineral and organic sources. This is consistent with findings from Kushwah et al. (2024) and Selim (2020), which show that integrated nutrient management enhances nutrient uptake, root growth, and soil microbial activity factors that contribute to improved plant architecture. Interesting, 40 kg N·ha^−1^ in combination with organic manure was sufficient to match or exceed the performance of the highest mineral N‐only treatment (80 kg N·ha^−1^), indicating that integrated fertilization can potentially reduce the required mineral N input without compromising crop performance. This is especially important for smallholder farmers, as it offers a cost‐effective and environmentally sustainable approach to maintaining high productivity under resource‐limited conditions (Bolo et al. 2024).

### 3.4 Effect of Organic Manure on Soil Matter Content

The results in Table 5 indicate that the application of both cattle and poultry manure significantly increased soil O.M content compared to unfertilized control. The highest O.M value (3.78%) was observed in the treatment receiving 5 t·ha^−1^ of cattle manure (C1B1A2), followed by the 10 t·ha^−1^ of cattle manure treatment (3.35%). In contrast, poultry manure treatments recorded comparatively lower O.M levels, with 2.64% for 5 t·ha^−1^ and 2.00% for 10 t·ha^−1^. The unfertilized control (C1B1A1) recorded the lowest O.M content (1.60%). These results suggest that cattle manure was more effective than poultry manure in enhancing soil O.M within the short time frame of the study. This may be attributed to the higher proportion of lignin and fibrous organic fractions in cattle manure, which decompose more slowly and contribute more to the accumulation of stable soil organic carbon (Bai et al. 2020; Rayne and Aula 2020). Interestingly, the lower application rate of cattle manure (5 t·ha^−1^) resulted in a slightly higher O.M content than the higher rate (10 t·ha^−1^), which may be explained by spatial variability, microbial decomposition dynamics, or incomplete decomposition of the bulkier organic matter during the first cropping season, an occurrence frequently reported in initial organic input applications (Ayamba et al. 2021).

**TABLE 5:** Soil organic matter content under different manure treatments.

Treatment	Manure	Rate applied (t·ha^−1^)	O.M (%)
C1B1A2	Cattle manure	5.00	3.78a
C1B1A3	Cattle manure	10.00	3.35a
C1B2A1	Poultry manure	5.00	2.64b
C1B3A1	Poultry manure	10.00	2.00c
C1B1A1	Control	0.00	1.60c

*Note:* The small letters (a, ab, b, c) indicate the statistical differences among the treatments.

Although poultry manure typically decomposes more rapidly and contains higher immediate nutrient concentrations, its lower O.M contribution in this study reflects its rapid mineralization rate, leaving fewer residual organic materials to contribute to stable carbon pools. This is particularly relevant in coarse‐textured or semi‐arid soils, such as those in Gaza province, where rapid organic matter turnover can limit long‐term carbon sequestration (Nahm 2003; Pellegrini et al. 2022). While some studies have reported similar trends in which cattle manure contributes more effectively to long‐term carbon accumulation than poultry manure (Singh et al. 2020; Xia et al. 2017), these effects are not universally consistent across all soil types and management systems, as decomposition rates and nutrient stabilization are strongly influenced by climate, soil texture, and application frequency (Yuan et al. 2023; Khan et al. 2025). The substantial difference in O.M content between control and manure‐treated plots emphasizes the critical role of organic amendments in enhancing soil quality in nutrient‐depleted systems (Singh et al. 2022). Therefore, integrating livestock‐based organic amendments, particularly cattle manure, represents a promising strategy to rebuild soil fertility and resilience in semi‐arid regions of sub‐Saharan Africa.

### 3.5 Effect of Organic Manure on Total Soil Nitrogen

The application of organic manures significantly influenced soil nitrogen content, as shown in Table 6. Among the treatments, the highest total N content (0.15%) was observed in plots receiving 10 t·ha^−1^ of poultry manure (C1B3A3), followed by 5 t·ha^−1^ of poultry manure (C1B2A1) with 0.11%. Both values were significantly higher than the unfertilized control (C1B1A1), which recorded 0.06%. Cattle manure also improved soil nitrogen content compared to the control, though to a lesser extent, with 0.09% and 0.08% for the 5 and 10 t·ha^−1^ applications, respectively. These findings underscore the superior nitrogen supplying capacity of poultry manure, likely due to its higher content of readily mineralizable nitrogen compounds, including uric acid, ammonium, and urea, which mineralize rapidly in soil (Adewole et al. 2016; Uwah et al. 2014). Rapid decomposition of poultry litter ensures quicker availability of nitrogen in forms readily assimilated by crops like maize, which have short growth cycles and high N demand in early development stages. In contrast, cattle manure contains more recalcitrant organic nitrogen, which tends to mineralize slowly, providing more sustained N release over long periods (Eckhardt et al. 2018; Zhang et al. 2015). This gradual release is beneficial in long‐term fertility management but less effective for meeting immediate crop N demand in short‐cycle crops.

**TABLE 6:** Total nitrogen content in soil after application of poultry and cattle manure.

Treatment	Manure	Rate applied (t·ha^−1^)	Total N (%)
C1B3A1	Poultry manure	10.00	0.15a
C1B2A1	Poultry manure	5.00	0.11b
C1B1A2	Cattle manure	5.00	0.09bc
C1B1A3	Cattle manure	10.00	0.08c
C1B1A1	Control	0.00	0.06c

*Note:* The small letters (a, ab, b, c) indicate the statistical differences among the treatments.

An interesting observation was that 5 t·ha^−1^ of poultry manure supplied more soil nitrogen than 10 t·ha^−1^ of cattle manure, reinforcing the concept that manure quality (i.e., nutrient composition and mineralization potential) is often more critical than quantity nitrogen availability (Palm et al. 2001). These results support current recommendations to evaluate organic resources based not only on application rates but also on their chemical characteristics, particularly when tailoring integrated nutrient management strategies for semi‐arid environments. The consistently low nitrogen content in the control treatment highlights the nutrient depleted nature of the soil and the critical role of organic amendments in improving soil fertility. As nitrogen remains one of the most limiting nutrients in tropical and sub‐tropical agroecosystems, the strategic use of high‐quality organic sources such as poultry manure can significantly reduce dependence on synthetic fertilizers and promote more sustainable crop production systems (Mutsaers et al. 2017; Vanlauwe et al. 2017).

### 3.6 Effect of Organic Manure on Soil Moisture Retention

Soil moisture content measured 21 days after irrigation was significantly affected by organic manure application (Table 7). The highest moisture retention (27.10%) was observed in the plot treated with 10 t·ha^−1^ of cattle manure, which was significantly higher than all other treatments. In contrast, treatments receiving 5 or 10 t·ha^−1^ of poultry manure and 5 t·ha^−1^ cattle manure showed similar moisture levels ranging between 24.06% and 24.71%, statistically comparable to the unfertilized control (25.21%). These findings suggest that higher application rates of cattle manure can meaningfully enhance the soil's water holding capacity. This improvement is likely due to cattle manure's higher content of fibrous and lignified organic material, which supports macroaggregate formation, increases soil porosity, and improves aggregate stability, all critical for retaining water within the soil matrix (Brempong et al. 2023; Kumar et al. 2022).

**TABLE 7:** Soil moisture retention 21 days after irrigation under different manure treatments.

Treatment	Manure	Rate applied (t·ha^−1^)	Moisture (%)
C1A3B1	Cattle manure	10.00	27.10a
C1B1A1	Control	0.00	25.21b
C1A1B3	Poultry manure	10.00	24.71b
C1B2A1	Poultry manure	5.00	24.11b
C1A2B1	Cattle manure	5.00	24.06b

*Note:* *p* value = 0.0003; CV = 1.92. The small letters (a, ab, b) indicate the statistical differences among the treatments.

Although poultry manure improved moisture conservation, its finer texture and higher decomposition rate may offer less structural benefit in the short term. This aligns with findings by Mateo‐Marín et al. (2022), and Mujdeci et al. (2017), who noted that coarser organic materials, such as those found in cattle manure, are more effective at improving physical properties like infiltration, field capacity, and moisture retention. Interesting, the unfertilized control plot showed moisture levels statistically like most of the manure treated plots, except for the 10 t·ha^−1^ cattle manure treatment. This suggests that a single application of organic manure may not be sufficient to generate significant physical changes in the soil within one cropping cycle, especially in semi‐arid or coarse‐textured soils. Nevertheless, the significant increase observed under the high‐rate cattle manure treatment underscores that even a single, adequately dosed application can yield measurable improvements in soil water retention early in the amendment timeline (Diacono and Montemurro 2011).

### 3.7 Economic Evaluation of Fertilization Treatments

The profitability of each fertilization treatment was evaluated using the Marginal Rate of Return (MRR), a critical indicator of economic viability, especially in smallholder contexts. According to Maselli et al. (2021), an MRR of 100% or higher is considered the minimum acceptable threshold to justify the adoption of a technology, meaning the investment returns at least double the cost. As shown in Table 8, organic manure treatments, particularly at moderate doses, outperformed mineral nitrogen in economic terms. The highest MRR (1020.50%) was observed with 5 t·ha^−1^ of poultry manure, suggesting that each unit of investment yielded a tenfold return compared to the control. Similarly, 5 t·ha^−1^ of cattle manure and 10 t·ha^−1^ poultry manure also showed excellent profitability, with MRRs of 989% and 863%, respectively. These results emphasize the cost‐effectiveness of moderate organic applications, particularly when organic resources are sourced locally and entail no acquisition cost.

**TABLE 8:** Economic evaluation.

Treatment	Grain yield (kg/ha)	Adjusted yield (kg/ha)	Gross benefits (Mt/ha)	Acquisition cost (Mt)	Transport cost (Mt/ha)	Application cost (Mt)	Total variable costs (Mt/ha)	Net benefits (Mt/ha)	MRR (%)
C1B1A3	3600	3240	48,600.00	0.00	500.00	2000.00	2500.00	46,100.00	369.80
C1B1A2	3940	3546	53,190.00	0.00	500.00	1000.00	1500.00	51,690.00	989.00
C1B3A1	3800	3420	51,300.00	0.00	500.00	1000.00	1500.00	49,800.00	863.00
C1B2A1	3560	3204	48,060.00	0.00	500.00	500.00	1000.00	47,060.00	1020.50
C1B1A1	2730	2457	36,855.00	0.00	0.00	0.00	0.00	36,855.00	Control
C2B1A1	4080	3672	55,080.00	3478.40	513.00	450.00	4441.00	50,638.80	310.40
C3B1A1	3880	3492	52,380.00	6956.40	513.00	600.00	8069.00	44,310.80	92.40

Abbreviations: MRR, Marginal Rate of Return; Mt, Meticais.

In contrast, mineral fertilizer treatments showed substantially lower returns. Although the 80 kg N·ha^−1^ application achieved relatively high maize yields, its MRR was only 92.40%, falling below the acceptability threshold. This low return is primarily due to the high cost of urea acquisition, transport, and application, which significantly reduced net benefits. The 40 kg N·ha^−1^ application, on the other hand, achieved a moderate MRR of 310.40%, indicating better economic efficiency. However, this still lagged the organic‐only options in terms of profitability. These findings align with studies by Jama et al. (2017) and Ismael et al. (2021), which reported that while mineral fertilizers can boost yields, their economic feasibility in low resource farming systems is often compromised by input costs, poor access, and variable nutrient use efficiency. Likewise, Maria et al. (2017) emphasized the relevance of ISFM strategies but noted that economic outcomes depend heavily on labor availability, input sourcing, and local price dynamics. Interestingly, in this study, treatments combining organic and mineral inputs did not surpass the profitability of organic only applications. This result is likely a consequence of the zero‐acquisition cost assumption for manure, which substantially reduced the total variable costs of organic treatments. In real world scenarios, if organic inputs must be purchased, the relative advantage may shift in favor of integrated systems. Nonetheless, these findings reinforce that locally sourced organic inputs when available can deliver competitive returns compared to synthetic fertilizers, especially in semi‐arid, smallholder systems like those in Gaza province.

## 4 Conclusion

This study assessed the agronomic and economic response of maize to integrated applications of organic and mineral nitrogen fertilization under smallholder farming conditions in Gaza province, Mozambique. The findings confirmed that combining poultry or cattle manure with nitrogen fertilizer significantly improved maize yield, plant growth, and soil fertility indicators compared to unfertilized controls or mineral nitrogen alone. Integrated treatments, particularly those combining poultry manure with 40–80 kg N·ha^−1^, produced the highest grain yields. Notably, the sole application of 5 t·ha^−1^ poultry manure emerged as the most economically viable strategy, achieving the highest marginal rate of return (MRR > 1000%) with minimal investment, making it a suitable option for resource‐constrained farmers. Additionally, organic amendments, especially cattle manure, contributed to improved soil moisture retention, a crucial factor for maintaining crop performance in semi‐arid environments. These findings reinforce the value of integrated fertilization in boosting both agronomic productivity and economic efficiency in maize‐based systems. However, the study was conducted over a single cropping season, which limits the assessment of long‐term soil fertility changes and cumulative nutrient effects. Future studies should examine the residual and seasonal impacts of integrated fertilization strategies on nutrient cycling, labor efficiency, and profitability across varied environmental conditions.

## Funding

This study was funded by the Alliance for a Green Revolution in Africa (AGRA).

## Conflicts of Interest

The authors declare no conflicts of interest.

## Supporting information


**Data S1:** Maize grain yield (t·ha^−1^).

## Data Availability

The data that support the findings of this study are available in the Supporting Information of this article.
