# Supplementary material for: Maize Yield and Economic Response to Integrated Organic and Nitrogen Fertilization in Gaza Province, Mozambique
Source: Plant Environ Interact. 2026 Jul 13;7(4):e70184. doi: 10.1002/pei3.70184 (PMC13358803; doi:10.1002/pei3.70184)
Supplement: Supplementary file 1 — Data S1: Maize grain yield (t·ha−1). [file PEI3-7-e70184-s001.docx]

**MAIZE GRAIN YIELD (t·ha^-1^)**

| **treat** | **bloc** | **yield** |  | **treat** | **bloc** | **yield** |  | **treat** | **bloc** | **yield** |
| --- | --- | --- | --- | --- | --- | --- | --- | --- | --- | --- |
| C1B3A3 | 1 | 5.60 |  | C2B3A3 | 1 | 6.06 |  | C3B2A1 | 1 | 4.96 |
| C1B3A3 | 2 | 5.16 |  | C2B3A3 | 2 | 4.24 |  | C3B2A1 | 2 | 5.81 |
| C1B3A3 | 3 | 5.27 |  | C2B3A3 | 3 | 4.04 |  | C3B2A1 | 3 | 4.93 |
| C1B2A3 | 1 | 4.64 |  | C2B2A3 | 1 | 4.54 |  | C3B3A1 | 1 | 4.93 |
| C1B2A3 | 2 | 4.21 |  | C2B2A3 | 2 | 4.69 |  | C3B3A1 | 2 | 5.26 |
| C1B2A3 | 3 | 4.04 |  | C2B2A3 | 3 | 6.10 |  | C3B3A1 | 3 | 5.47 |
| C1B1A3 | 1 | 3.15 |  | C2B1A3 | 1 | 3.60 |  | C3B1A2 | 1 | 5.20 |
| C1B1A3 | 2 | 3.20 |  | C2B1A3 | 2 | 4.50 |  | C3B1A2 | 2 | 4.65 |
| C1B1A3 | 3 | 4.46 |  | C2B1A3 | 3 | 5.33 |  | C3B1A2 | 3 | 3.92 |
| C1B3A2 | 1 | 2.72 |  | C2B3A2 | 1 | 4.21 |  | C3B1A1 | 1 | 3.38 |
| C1B3A2 | 2 | 2.85 |  | C2B3A2 | 2 | 3.10 |  | C3B1A1 | 2 | 3.41 |
| C1B3A2 | 3 | 2.14 |  | C2B3A2 | 3 | 2.84 |  | C3B1A1 | 3 | 4.86 |
| C1B2A2 | 1 | 2.57 |  | C2B2A2 | 1 | 3.70 |  | C3B2A2 | 1 | 2.80 |
| C1B2A2 | 2 | 3.40 |  | C2B2A2 | 2 | 4.06 |  | C3B2A2 | 2 | 6.14 |
| C1B2A2 | 3 | 3.37 |  | C2B2A2 | 3 | 3.74 |  | C3B2A2 | 3 | 3.96 |
| C1B1A2 | 1 | 3.31 |  | C2B1A2 | 1 | 5.40 |  | C3B1A3 | 1 | 3.39 |
| C1B1A2 | 2 | 4.03 |  | C2B1A2 | 2 | 5.09 |  | C3B1A3 | 2 | 4.87 |
| C1B1A2 | 3 | 4.47 |  | C2B1A2 | 3 | 5.15 |  | C3B1A3 | 3 | 4.91 |
| C1B3A1 | 1 | 2.86 |  | C2B3A1 | 1 | 4.39 |  | C3B2A3 | 1 | 4.57 |
| C1B3A1 | 2 | 4.36 |  | C2B3A1 | 2 | 4.13 |  | C3B2A3 | 2 | 4.49 |
| C1B3A1 | 3 | 4.19 |  | C2B3A1 | 3 | 5.73 |  | C3B2A3 | 3 | 4.18 |
| C1B2A1 | 1 | 3.65 |  | C2B2A1 | 1 | 2.26 |  | C3B3A3 | 1 | 3.63 |
| C1B2A1 | 2 | 3.55 |  | C2B2A1 | 2 | 4.33 |  | C3B3A3 | 2 | 4.52 |
| C1B2A1 | 3 | 3.48 |  | C2B2A1 | 3 | 5.45 |  | C3B3A3 | 3 | 4.43 |
| C1B1A1 | 1 | 2.48 |  | C2B1A1 | 1 | 3.37 |  | C3B3A2 | 1 | 3.21 |
| C1B1A1 | 2 | 3.17 |  | C2B1A1 | 2 | 4.71 |  | C3B3A2 | 2 | 6.03 |
| C1B1A1 | 3 | 2.54 |  | C2B1A1 | 3 | 4.18 |  | C3B3A2 | 3 | 4.03 |

**CROP HEIGHT (cm)**

| **treat** | **bloc** | **Crop height** |  | **treat** | **bloc** | **Crop height** |  | **treat** | **bloc** | **Crop height** |
| --- | --- | --- | --- | --- | --- | --- | --- | --- | --- | --- |
| C1B3A3 | 1 | 175 |  | C2B3A3 | 1 | 221 |  | C3B2A1 | 1 | 208 |
| C1B3A3 | 2 | 218 |  | C2B3A3 | 2 | 207 |  | C3B2A1 | 2 | 219 |
| C1B3A3 | 3 | 226 |  | C2B3A3 | 3 | 184 |  | C3B2A1 | 3 | 205 |
| C1B2A3 | 1 | 188 |  | C2B2A3 | 1 | 207 |  | C3B3A1 | 1 | 224 |
| C1B2A3 | 2 | 205 |  | C2B2A3 | 2 | 223 |  | C3B3A1 | 2 | 226 |
| C1B2A3 | 3 | 218 |  | C2B2A3 | 3 | 210 |  | C3B3A1 | 3 | 227 |
| C1B1A3 | 1 | 180 |  | C2B1A3 | 1 | 208 |  | C3B1A2 | 1 | 226 |
| C1B1A3 | 2 | 203 |  | C2B1A3 | 2 | 198 |  | C3B1A2 | 2 | 327 |
| C1B1A3 | 3 | 210 |  | C2B1A3 | 3 | 220 |  | C3B1A2 | 3 | 199 |
| C1B3A2 | 1 | 158 |  | C2B3A2 | 1 | 174 |  | C3B1A1 | 1 | 208 |
| C1B3A2 | 2 | 179 |  | C2B3A2 | 2 | 182 |  | C3B1A1 | 2 | 219 |
| C1B3A2 | 3 | 184 |  | C2B3A2 | 3 | 204 |  | C3B1A1 | 3 | 205 |
| C1B2A2 | 1 | 173 |  | C2B2A2 | 1 | 187 |  | C3B2A2 | 1 | 196 |
| C1B2A2 | 2 | 205 |  | C2B2A2 | 2 | 199 |  | C3B2A2 | 2 | 225 |
| C1B2A2 | 3 | 198 |  | C2B2A2 | 3 | 214 |  | C3B2A2 | 3 | 186 |
| C1B1A2 | 1 | 169 |  | C2B1A2 | 1 | 206 |  | C3B3A2 | 1 | 197 |
| C1B1A2 | 2 | 211 |  | C2B1A2 | 2 | 217 |  | C3B3A2 | 2 | 218 |
| C1B1A2 | 3 | 224 |  | C2B1A2 | 3 | 207 |  | C3B3A2 | 3 | 210 |
| C1B3A1 | 1 | 189 |  | C2B3A1 | 1 | 203 |  | C3B1A3 | 1 | 206 |
| C1B3A1 | 2 | 212 |  | C2B3A1 | 2 | 226 |  | C3B1A3 | 2 | 216 |
| C1B3A1 | 3 | 208 |  | C2B3A1 | 3 | 222 |  | C3B1A3 | 3 | 188 |
| C1B2A1 | 1 | 193 |  | C2B2A1 | 1 | 193 |  | C3B2A3 | 1 | 206 |
| C1B2A1 | 2 | 190 |  | C2B2A1 | 2 | 211 |  | C3B2A3 | 2 | 234 |
| C1B2A1 | 3 | 214 |  | C2B2A1 | 3 | 216 |  | C3B2A3 | 3 | 211 |
| C1B1A1 | 1 | 161 |  | C2B1A1 | 1 | 177 |  | C3B3A3 | 1 | 210 |
| C1B1A1 | 2 | 181 |  | C2B1A1 | 2 | 197 |  | C3B3A3 | 2 | 216 |
| C1B1A1 | 3 | 189 |  | C2B1A1 | 3 | 199 |  | C3B3A3 | 3 | 195 |

**DRY MASS YIELD (t·ha^-1^)**

| **treat** | **bloc** | **Crop height** |  | **treat** | **bloc** | **Crop height** |  | **treat** | **bloc** | **Crop height** |
| --- | --- | --- | --- | --- | --- | --- | --- | --- | --- | --- |
| C1B3A3 | 1 | 10.44 |  | C2B3A3 | 1 | 14.25 |  | C3B2A1 | 1 | 12.75 |
| C1B3A3 | 2 | 11.78 |  | C2B3A3 | 2 | 10.72 |  | C3B2A1 | 2 | 12.53 |
| C1B3A3 | 3 | 8.91 |  | C2B3A3 | 3 | 15.13 |  | C3B2A1 | 3 | 6.94 |
| C1B2A3 | 1 | 9.31 |  | C2B2A3 | 1 | 11.81 |  | C3B3A1 | 1 | 13.75 |
| C1B2A3 | 2 | 10.50 |  | C2B2A3 | 2 | 13.09 |  | C3B3A1 | 2 | 11.31 |
| C1B2A3 | 3 | 14.00 |  | C2B2A3 | 3 | 13.47 |  | C3B3A1 | 3 | 13.13 |
| C1B1A3 | 1 | 7.75 |  | C2B1A3 | 1 | 10.09 |  | C3B1A2 | 1 | 15.03 |
| C1B1A3 | 2 | 11.50 |  | C2B1A3 | 2 | 7.06 |  | C3B1A2 | 2 | 10.28 |
| C1B1A3 | 3 | 10.28 |  | C2B1A3 | 3 | 11.94 |  | C3B1A2 | 3 | 11.22 |
| C1B3A2 | 1 | 12.84 |  | C2B3A2 | 1 | 9.25 |  | C3B1A1 | 1 | 14.00 |
| C1B3A2 | 2 | 9.22 |  | C2B3A2 | 2 | 10.19 |  | C3B1A1 | 2 | 9.94 |
| C1B3A2 | 3 | 9.56 |  | C2B3A2 | 3 | 8.84 |  | C3B1A1 | 3 | 7.03 |
| C1B2A2 | 1 | 13.88 |  | C2B2A2 | 1 | 10.91 |  | C3B2A2 | 1 | 11.53 |
| C1B2A2 | 2 | 10.66 |  | C2B2A2 | 2 | 15.19 |  | C3B2A2 | 2 | 7.09 |
| C1B2A2 | 3 | 7.38 |  | C2B2A2 | 3 | 12.78 |  | C3B2A2 | 3 | 6.31 |
| C1B1A2 | 1 | 12.66 |  | C2B1A2 | 1 | 12.63 |  | C3B3A2 | 1 | 13.72 |
| C1B1A2 | 2 | 12.06 |  | C2B1A2 | 2 | 11.19 |  | C3B3A2 | 2 | 8.06 |
| C1B1A2 | 3 | 16.75 |  | C2B1A2 | 3 | 10.13 |  | C3B3A2 | 3 | 8.38 |
| C1B3A1 | 1 | 16.41 |  | C2B3A1 | 1 | 13.16 |  | C3B1A3 | 1 | 10.66 |
| C1B3A1 | 2 | 6.75 |  | C2B3A1 | 2 | 8.81 |  | C3B1A3 | 2 | 10.72 |
| C1B3A1 | 3 | 9.00 |  | C2B3A1 | 3 | 9.88 |  | C3B1A3 | 3 | 13.06 |
| C1B2A1 | 1 | 9.25 |  | C2B2A1 | 1 | 11.84 |  | C3B2A3 | 1 | 10.56 |
| C1B2A1 | 2 | 7.00 |  | C2B2A1 | 2 | 8.06 |  | C3B2A3 | 2 | 12.69 |
| C1B2A1 | 3 | 6.38 |  | C2B2A1 | 3 | 11.81 |  | C3B2A3 | 3 | 11.63 |
| C1B1A1 | 1 | 11.13 |  | C2B1A1 | 1 | 8.34 |  | C3B3A3 | 1 | 9.31 |
| C1B1A1 | 2 | 11.22 |  | C2B1A1 | 2 | 9.78 |  | C3B3A3 | 2 | 8.09 |
| C1B1A1 | 3 | 7.00 |  | C2B1A1 | 3 | 8.34 |  | C3B3A3 | 3 | 12.81 |
